# Triglyceride Glucose Index Is Associated With Arterial Stiffness and 10-Year Cardiovascular Disease Risk in a Chinese Population

**DOI:** 10.3389/fcvm.2021.585776

**Published:** 2021-03-19

**Authors:** Wen Guo, Wenfang Zhu, Juan Wu, Xiaona Li, Jing Lu, Pei Qin, Cheng Zhu, Nianzhen Xu, Qun Zhang

**Affiliations:** Department of Health Promotion Center, The First Affiliated Hospital With Nanjing Medical University, Nanjing, China

**Keywords:** triglyceride glucose index, arterial stiffness, Framingham risk score, brachial-ankle pulse wave velocity, insulin resistance

## Abstract

**Background:** Insulin resistance (IR) is a significant risk factor for cardiovascular disease (CVD). In this study, the association of the triglyceride glucose (TyG) index, a simple surrogate marker of IR, with arterial stiffness and 10-year CVD risk was evaluated.

**Methods:** A total of 13,706 participants were enrolled. Anthropometric and cardiovascular risk factors were determined in all participants, while serum insulin levels were only measured in 955 participants. Arterial stiffness was measured through brachial-ankle pulse wave velocity (baPWV), and 10-year CVD risk was evaluated using the Framingham risk score.

**Results:** All participants were classified into four groups according to the quartile of the TyG index. BaPWV and the percentage of participants in the 10-year CVD risk categories significantly increased with increasing quartiles of the TyG index. Logistic regression analysis showed that the TyG index was independently associated with a high baPWV and 10-year CVD risk after adjusting for traditional CVD risk factors. The area under the receiver operating characteristics curve (AUROC) of the TyG index for predicting a high baPWV was 0.708 (95%*CI* 0.693–0.722, *P* < 0.001) in women, higher than that in men. However, the association of the homeostatic model assessment of IR (HOMA-IR) with a high baPWV and the 10-year CVD risk was absent when adjusting for multiple risk factors in 955 participants.

**Conclusions:** The TyG index is independently associated with arterial stiffness and 10-year CVD risk.

## Introduction

Cardiovascular disease (CVD) is a leading cause of death among middle-aged and elderly individuals in both developing and developed countries (1). Stroke and ischemic heart disease were the leading causes of death and disability-adjusted life-years at the national level in China in 2017, as estimated by the Global Burden of Disease Study (2). Therefore, early identification of individuals with a higher risk of CVD and the early prevention of the disease are essential approaches to solving the public health problem.

Insulin resistance (IR) plays a vital role in the physiopathologic mechanism of type 2 diabetes mellitus (T2DM). IR is a hallmark of metabolic syndrome (MetS) and non-alcoholic fatty liver disease (NAFLD) (3). Recently, a growing body of evidences have demonstrated that IR is a critical risk factor for CVD (4, 5). Several studies have reported that the triglyceride-glucose (TyG) index is significantly correlated with IR, as evaluated through hyperinsulinemic-euglycemic clamp testing or homeostatic model assessment of IR (HOMA-IR) (6, 7). Thus, the TyG index has been considered to be a reliable surrogate marker of IR. Some recent studies have shown that the TyG index is not only correlated with metabolic diseases but is also associated with CVD (8, 9). Cross-sectional observational investigations among Korean adults and Greek postmenopausal women both indicate that the TyG index is independently associated with increased arterial stiffness assessed through brachial-ankle pulse wave velocity (baPWV) (10, 11). However, there is a paucity of data on the relationship between the TyG index and baPWV, which has an independent prognostic value for the risk of cardiovascular events in a Chinese population. Therefore, we investigated the association of the TyG index with arterial stiffness in Chinese adults using baPWV and Framingham risk score, a widely used metric for estimating the global risk of a 10-year CVD event.

## Materials and Methods

### Study Population

A total of 14,233 participants who participated in a comprehensive health examination including baPWV measurement were selected from the Health Promotion Center of the First Affiliated Hospital of Nanjing Medical University from September 2017 and December 2019. Subjects with previous history of cardiovascular events, cerebrovascular accident, diabetes, malignancy, systemic acute or chronic inflammatory diseases such as chronic nephritis, or liver, kidney, or other diseases associated with lipid metabolism disorders such as hypothyroidism were excluded from the study. We also excluded subjects taking statins or triglyceride-lowering medications (fenofibrate or omega-3). After applying the exclusion criteria, a total of 13,706 participants were enrolled in our final analysis. We retrospectively analyzed clinical data of 13,706 participants and found that only 955 participants performed insulin measurement. Written consents were obtained from all participants. This study was approved by the Human Research Ethics Committee of the First Affiliated Hospital of Nanjing Medical University. All procedures performed in the studies were done in accordance with the ethical standards of the institutional research committee and with the Declaration of Helsinki (as revised in Brazil 2013).

### Physical Examination and Biochemical Tests

We measured the weight and height of all participants and calculated the body mass index (BMI, kg/m^2^). Experienced technicians used an automated blood pressure (BP) monitor (HEM-7080IC; Omron Healthcare, Lake Forest, IL, USA) to measure systolic and diastolic blood pressures (SBP, DBP) after a 5-min rest period with all participants' arm placed at heart level. A standard questionnaire was used to evaluate smoking habit of the participants, history of acute and chronic illnesses, and drug use. Venous blood samples were collected after overnight fasting. Routine biochemical analyses, including serum lipids, glucose, and uric acid, were measured using enzymatic methods (Chemistry Analyzer Hitachi 7020, Japan). Glycated hemoglobin A1c (HbA1c) values were measured through a high-performance liquid chromatography, and plasma insulin concentration was determined using a chemiluminescence-based assay (Daiichi, Japan).

### Measurement of baPWV

After resting for 15–30 min, the baPWV values of participants were measured using a VP-1000 automated PWV/ABI analyzer. Briefly, baPWV was measured from the ascending point of the right brachial pulse volume recorder to the ascending point of each ankle pulse volume recorder, as described previously (12). The average values of the left and right ba-PWV were used for analysis. In this study, high baPWV was defined as a value higher than the cut-off level between the third and fourth quartiles (>75th percentile) of baPWV, which was 1,460 cm/s for all participants, 1,486 cm/s for men, and 1,419.5 cm/s for women.

### Framingham 10-Year Risk Estimation

Framingham risk score was used to predict the patients' risk of CVD for up to 10 years after estimation. The Framingham risk score was calculated based on variables including age, sex, total cholesterol (TC), high-density lipoprotein cholesterol (HDL-C), systolic blood pressure (SBP) (treated or untreated), and smoking habits in men and women, separately (13). The estimated risk for 10-year CVD was classified as low risk (<10%), intermediate risk (10–20%), and high risk (≥20%) (14).

### Calculations

HOMA-IR = fasting blood glucose (mmol/L) × fasting insulin (uIU/mL) / 22.5; BMI = weight (kg) / height (m)^2^; TyG index = ln [fasting triglycerides (mg/dL) × fasting glucose (mg/dL)/2]; Pulse pressure (PP) = SBP- diastolic blood pressure (DBP).

### Statistical Analysis

Continuous variables were expressed as mean ± standard deviation (SD). Differences among the four groups were tested using a one-way analysis of variance (ANOVA) with Bonferroni correction for pairwise comparisons. Categorical variables were expressed as percentages (numbers) and analyzed using the Chi-square test. Age-adjusted baPWV means and standard errors were calculated using ANCOVA based on TyG quartiles. The correlations between the parameters were analyzed using Pearson's correlation. Binary logistic regression analysis was performed to determine the association of the TyG index with high baPWV (0 = normal baPWV, 1 = high baPWV) and 10-year CVD risk (0 = low risk, 1 = intermediate or high risk). Sex-interaction between the TyG index and baPWV were performed using generalized linear models (GLMs). Receiver operator characteristic (ROC) analyses were performed to calculate the area under the ROC curve (AUC) of the TyG index for incident high baPWV. Data were analyzed using SPSS18.0 statistical software, with statistical significance defined as *p* < 0.05 (two-sided).

## Results

### Baseline Characteristics of the Study Population

The clinical and biochemical characteristics of the participants are listed in Table 1. The participants were classified into four groups based on their TyG index levels. Age, the proportion of men, smoking, and the prevalence of hypertension were more likely to increase across the quartiles of the TyG index. Metabolic parameters, including BMI, SBP, DBP, PP, fasting plasma glucose (FBG), HbA1c TC, triglyceride (TG), low density lipoprotein-cholesterol (LDL-C), and uric acid, increased, and the level of HDL-C decreased in proportion to the TyG index quartiles.

**Table 1:** Clinical characteristics of the study population according to TyG index.

	**Q1 (lowest)**	**Q2**	**Q3**	**Q4 (highest)**	***P*-value**
	(6.82–8.34)	(8.35–8.70)	(8.71–9.13)	(9.14–12.23)	
N	3,473	3,380	3,458	3,395	
Age (years)	47.12 ± 10.45	49.86 ± 9.71	50.46 ± 9.80	50.24 ± 9.37	<0.01
Sex (male/Female)	1,223/2,250	1,733/1,647	2,140/1,318	2,537/858	<0.01
Smoking (%)	343 (9.9)	509 (15.1)	764 (22.1)	980 (28.9)	<0.01
BMI (kg/m^2^)	22.76 ± 2.81	24.17 ± 2.90	25.14 ± 2.91	26.32 ± 3.11	<0.01
SBP (mmHg)	120.27 ± 16.49	125.69 ± 16.85	128.54 ± 16.94	132.38 ± 16.76	<0.01
DBP (mmHg)	72.65 ± 10.73	76.72 ± 10.76	78.90 ± 10.93	82.30 ± 10.92	<0.01
PP (mmHg)	47.62 ± 10.84	48.97 ± 11.51	49.64 ± 11.56	50.10 ± 11.75	<0.01
FBG (mmol/L)	5.01 ± 0.45	5.27 ± 0.59	5.49 ± 0.78	6.33 ± 2.03	<0.01
HbA1c (%)	5.38 ± 0.37	5.51 ± 0.43	5.62 ± 0.56	6.03 ± 1.15	<0.01
TC (mmol/L)	5.01 ± 0.93	5.28 ± 0.98	5.50 ± 1.03	5.73 ± 1.16	<0.01
TG (mmol/L)	0.81 ± 0.16	1.22 ± 0.17	1.72 ± 0.28	3.37 ± 1.91	<0.01
LDL-C (mmol/L)	3.00 ± 0.68	3.29 ± 0.72	3.49 ± 0.75	3.57 ± 0.81	<0.01
HDL-C (mmol/L)	1.54 ± 0.32	1.40 ± 0.28	1.28 ± 0.25	1.17 ± 0.23	<0.01
TyG index	8.06 ± 0.21	8.53 ± 0.10	8.90 ± 0.12	9.60 ± 0.43	<0.01
Uric acid (μmol/L)	290.32 ± 73.99	320.48 ± 78.82	345.64 ± 81.62	379.96 ± 86.85	<0.01
Creatinine (μmol/L)	63.15 ± 13.24	67.16 ± 14.44	69.39 ± 14.28	71.15 ± 14.95	<0.01
Hypertension, *n* (%)	299 (8.61)	521 (15.41)	718 (20.76)	982 (28.92)	<0.01
Anti-hypertensive drugs, *n* (%)	284 (8.17)	509 (15.06)	699 (20.21)	954 (28.10)	<0.01

*Values are presented as mean ± standard deviation*.

*BMI, body mass index; SBP, systolic blood pressure; DBP, diastolic blood pressure; PP, Pulse pressure; FBG, fasting blood glucose; TC, total cholesterol; TG, triacylglyceride; LDL-C, high-density lipoprotein cholesterol; HDL-C, high-density lipoprotein cholesterol*.

### baPWV and 10-Year CVD Risk Compared Across the TyG Index Quartiles

**Figure 1:**
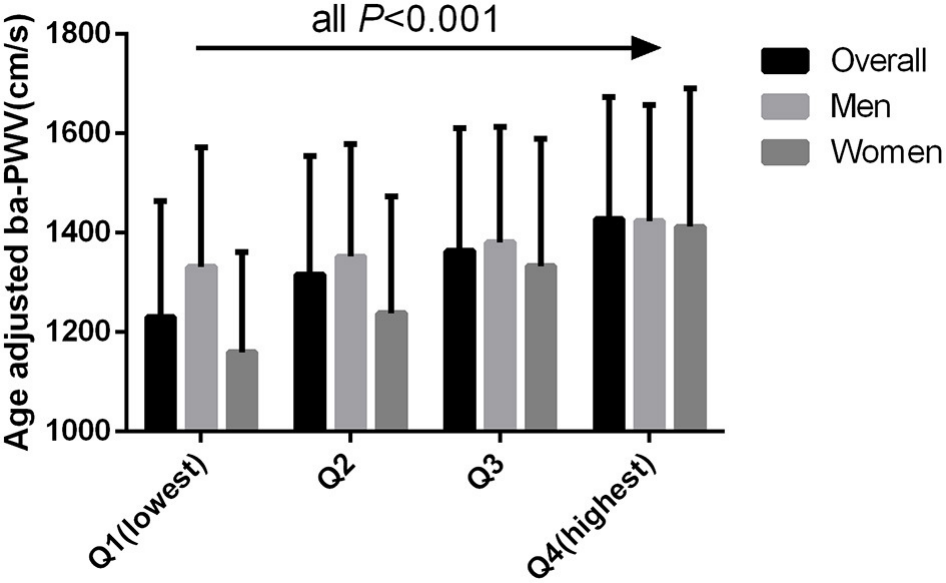
Age adjusted BaPWV compared across the TyG index quartiles for all participants, men, and women.

**Figure 2:**
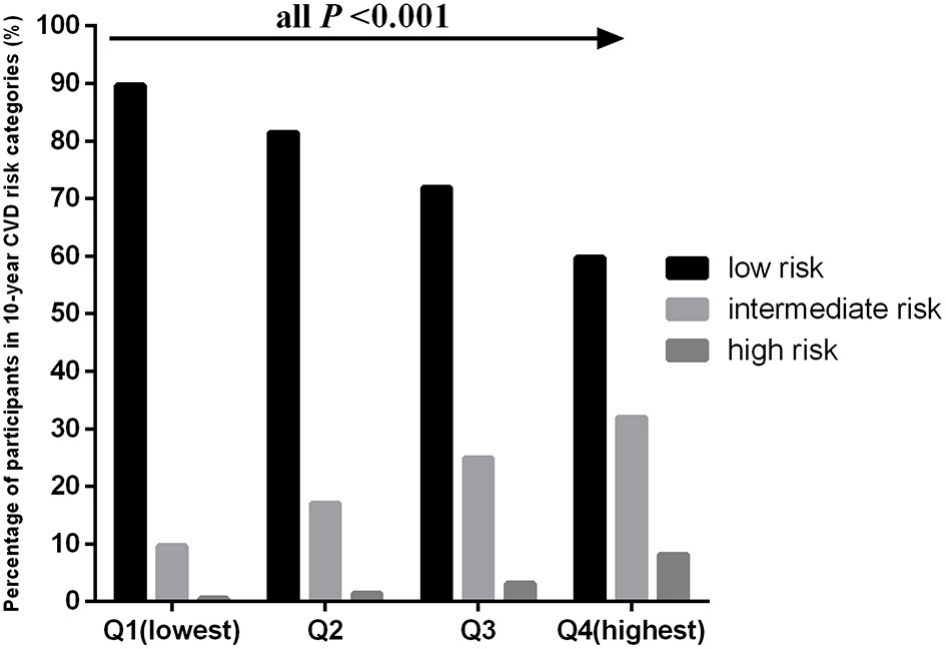
Percentages of 10-CVD risk categorize compared across the quartiles of the TyG index.

The age-adjusted mean baPWV significantly increased with increasing quartiles of the TyG index in the overall population (1,230.31 ± 234.02 vs. 1,316.48 ± 238.39 vs. 1,363.55 ± 247.60 vs. 1,427.39 ± 246.14 cm/s, *P* < 0.001). This phenomenon was also observed in men (1,331.43 ± 240.31 vs. 1,352.63 ± 226.16 vs. 1,380.95 ± 232.22 vs. 1,423.77 ± 233.44 cm/s, *P* < 0.001) and women (1,159.79 ± 201.91 vs. 1,238.22 ± 234.69 vs. 1,322.67 ± 257.05 vs. 1,412.62 ± 278.40 cm/s, *P* < 0.001) (Figure 1). Figure 2 indicated that the highest prevalence of participants with low or intermediate TyG index were in the low and intermediate risks of CVD, whereas the participants with high TyG index were the most prevalent in the groups with high CVD risk.

### Relationships Between baPWV, Framingham Risk Score, and Clinical Variables

Table 2 showed that baPWV was significantly correlated with age, BMI, SPB, DBP, PP, FBG, HbA1c, TC, TG, LDL-C, HDL-C, TyG index, and uric acid. The relationships between Framingham risk score and clinical variables, including TyG index, were parallel to the associations between baPWV and the clinical variables.

**Table 2:** Correlation between ba-PWV, Framingham risk score, and clinical variables.

	**ba-PWV**		**Framingham risk score**	
	***r***	***P***	***r***	***P***
Age (years)	0.542	<0.001	0.435	<0.001
BMI (kg/m^2^)	0.175	<0.001	0.281	<0.001
SBP (mmHg)	0.622	<0.001	0.352	<0.001
DBP (mmHg)	0.486	<0.001	0.332	<0.001
PP (mmHg)	0.458	<0.001	0.203	<0.001
FBG (mmol/L)	0.260	<0.001	0.231	<0.001
HbA1c (%)	0.269	<0.001	0.238	<0.001
TC (mmol/L)	0.104	<0.001	0.175	<0.001
TG (mmol/L)	0.163	<0.001	0.287	<0.001
LDL-C (mmol/L)	0.110	<0.001	0.207	<0.001
HDL-C (mmol/L)	−0.112	<0.001	−0.302	<0.001
TyG index	0.283	<0.001	0.362	<0.001
Uric acid (μmol/L)	0.166	<0.001	0.331	<0.001

*BMI, body mass index; SBP, systolic blood pressure; DBP, diastolic blood pressure; PP, Pulse pressure; FBG, fasting blood glucose; TC, total cholesterol; TG, triacylglyceride; LDL-C, high-density lipoprotein cholesterol; HDL-C, high-density lipoprotein cholesterol*.

### Relationship Between High baPWV and the TyG Index Through Binary Logistic Regression Analysis

After adjusting for other risk factors, including age, smoking, BMI, pulse pressure, HbA1c, TC, LDL-C, HDL-C, uric acid, and antihypertensive medication status, the TyG index was found to be an independent risk factor for high baPWV in all participants (*OR* = 1.514, 95%*CI* 1.371–1.672, *P* < 0.001). Both in men (*OR* = 1.531, 95%*CI* 1.348–1.739, *P* < 0.001) and women (*OR* = 1.848, 95%*CI* 1.586–2.189, *P* < 0.001), this relationship remained statistically significant after adjusting for confounding variables (Table 3).

**Table 3:** Logistic regression analysis of the association between high baPWV and the TyG index.

**Model**	**Overall**		**Men**		**Women**	
	**OR (95%*CI*)**	***P***	**OR (95%*CI*)**	***P***	**OR (95%*CI*)**	***P***
1	2.137(2.006–2.276)	<0.001	1.561(1.438–1.694)	<0.001	3.747(3.340–4.204)	<0.001
2	1.514(1.371–1.672)	<0.001	1.531(1.348–1.739)	<0.001	1.848(1.586–2.189)	<0.001

*Model1: unadjusted*.

*Model2: adjustment for age, smoking, BMI, pulse pressure, HbA1c, TC, LDL-C, HDL-C, uric acid, and antihypertensive medication status*.

### Relationship Between the 10-Year CVD Risk and the TyG Index Through Binary Logistic Regression Analysis

The TyG index was an independent risk factor for intermediate or high risk in all participants (*OR* = 1.420, 95%*CI* 1.147–1.756, *P* <0.001), men (*OR* = 1.323 95%*CI* 1.056–1.657, *P* = 0.015), and women (*OR* = 2.345, 95%*CI* 1.179–4.667, *P* = 0.015), respectively, after adjusting for multiple risk factors, including BMI, pulse pressure, HbA1c, LDL-C, and uric acid (Table 4).

**Table 4:** Logistic regression analysis of the association between the 10-year CVD risk and TyG index.

**Model**	**Overall**		**Men**		**Women**	
	**OR (95%*CI*)**	***P***	**OR (95%*CI*)**	***P***	**OR (95%*CI*)**	***P***
1	2.803 (2.623–2.996)	<0.001	1.875 (1.737–2.025)	<0.001	3.954 (3.078–5.078)	<0.001
2	1.420 (1.147–1.756)	<0.001	1.323 (1.056–1.657)	0.015	2.345 (1.179–4.667)	0.015

*Model1: unadjusted*.

*Model2: adjustment for BMI, pulse pressure, HbA1c, LDL-C, and uric acid*.

### Gender Differences in the TyG Index and High baPWV

The TyG index was positively correlated with high baPWV (*OR* = 1.567, 95%*CI* 1.448–1.697, *P* < 0.001) and The TyG index × Sex was also positively correlated with high baPWV (*OR* = 2.226, 95%*CI* 1.966–2.613, *P* < 0.001) in all participants. Meanwhile, we also found that there was a significant sex-interaction between the TyG index and baPWV (*F* = 69.92, *P* < 0.001).

### Diagnostic Value of the TyG Index for Predicting High baPWV

**Figure 3:**
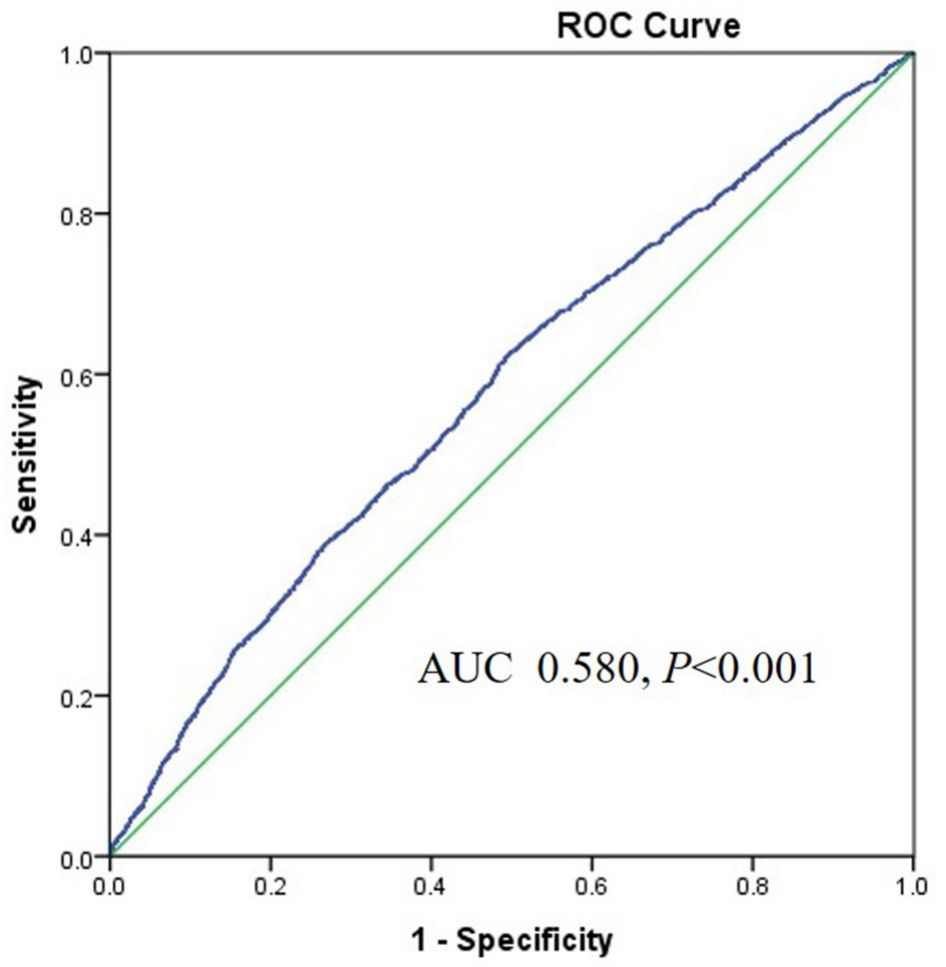
The area under the ROC (AUROC) of the TyG index for predicting high baPWV in women.

**Figure 4:**
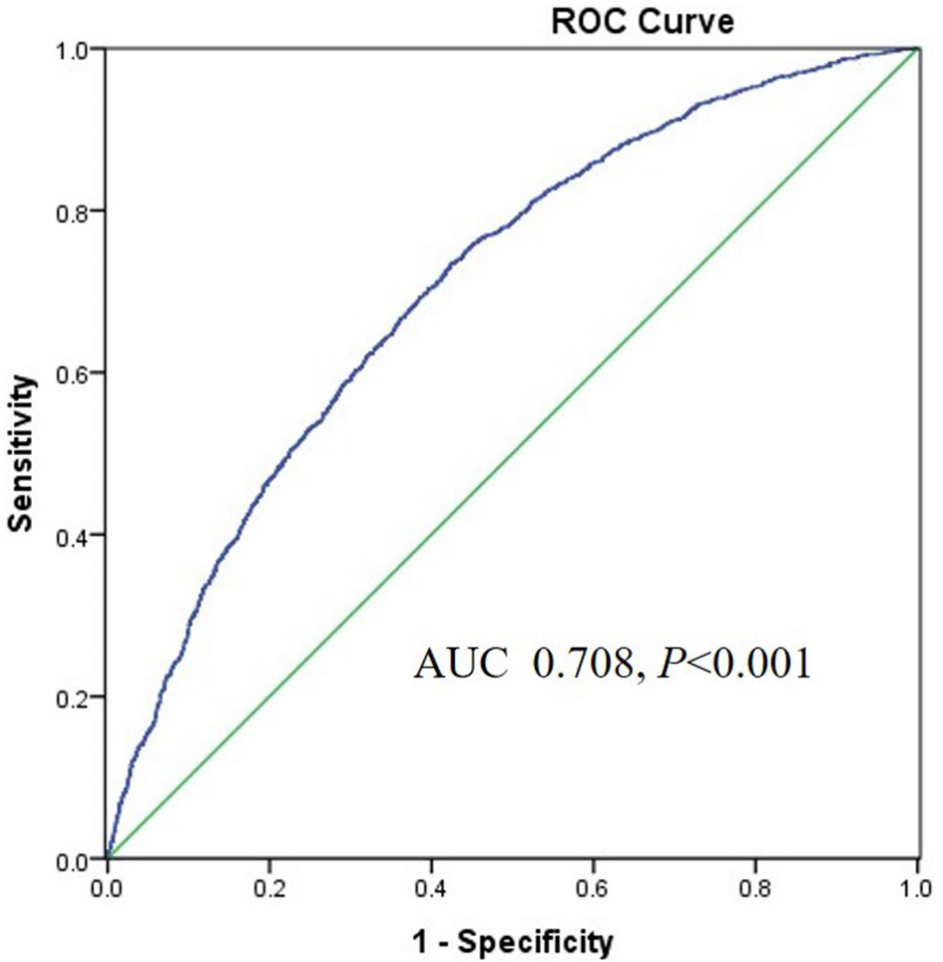
The area under the ROC (AUROC) of the TyG index for predicting high baPWV in men.

The area under the ROC (AUROC) of the TyG index for predicting high baPWV was 0.708 (95%*CI* 0.693–0.722, *P* < 0.001) in women and the optimal cut-off point for the TyG was 8.55 (sensitivity: 70.4 %, specificity: 60.4%) (Figure 3). The AUC value of the TyG index [0.580 (95%*CI* 0.565–0.595), *P* < 0.001] for predicting high baPWV in men was relatively smaller than that in women (Figure 4), which indicated that the TyG index was an acceptable predictor of high baPWV only in women.

### Diagnostic Value of the TyG Index for Predicting the 10-Year CVD Risk

**Figure 5:**
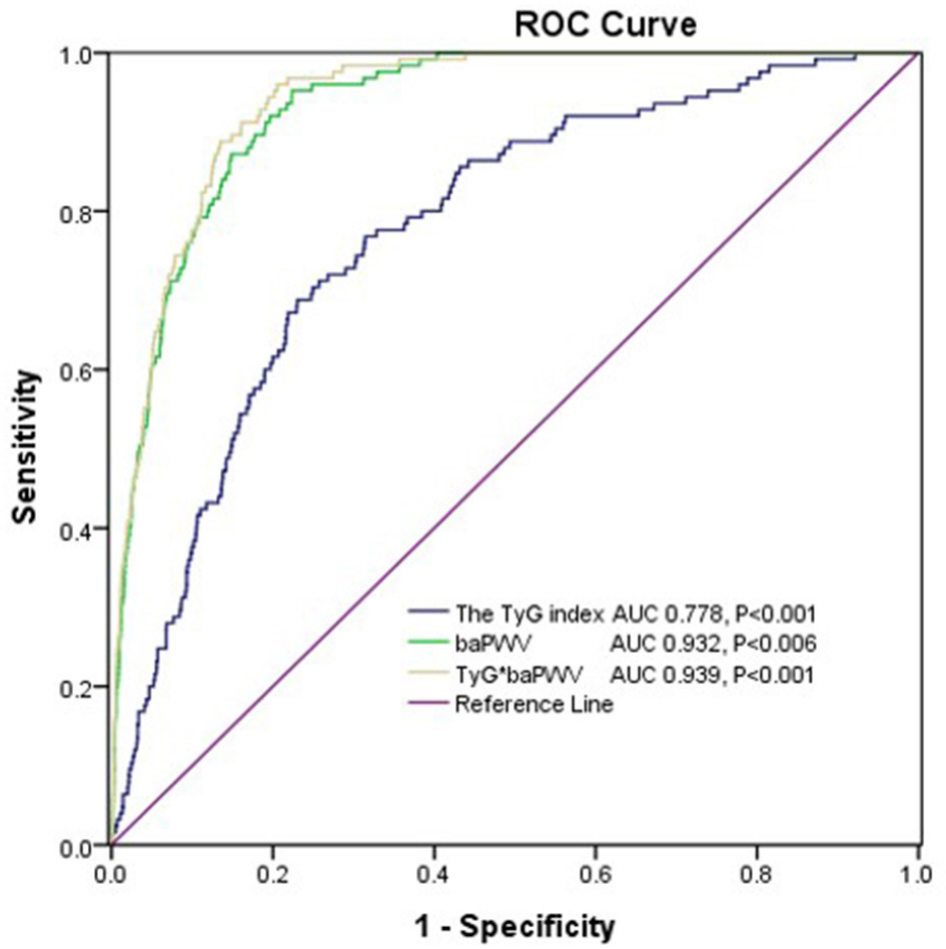
The areas under the ROC (AUROCs) of the TyG index, baPWV, and TyG-baPWV for predicting 10-CVD risk in women.

The AUROC of the TyG index for predicting the 10-year CVD risk was 0.778 (95%*CI* 0.739–0.816, *P* < 0.001) in women and the optimal cut-off point for the TyG index was 8.84 (sensitivity: 71.2 %, specificity: 74.3%). The AUC value of the TyG index [0.609 (95%*CI* 0.596–0.621), *P* < 0.001] for predicting 10-year CVD risk in men was relatively smaller than that in women, which indicated that the TyG index was an acceptable predictor of 10-year CVD risk only in women. The AUROCs of the baPWV and TyG-baPWV for predicting the 10-year CVD risk were 0.932 (95%*CI* 0.917–0.947, *P* < 0.001) and 0.939 (95%*CI* 0.926–0.953, *P* < 0.001) in women, respectively, which were both larger than that of the TyG index (Figure 5).

### Associations of IR Markers With baPWV and the 10-Year CVD Risk

In our study, the plasma insulin level was determined, and HOMA-IR was calculated in 955 participants. We analyzed and compared the associations of IR markers, including HOMA-IR, and the TyG index with baPWV and the 10-year CVD risk, respectively, in these 955 participants. Pearson's correlation analysis showed that baPWV was positively correlated with HOMA-IR (*r* = 0.147, *P* < 0.001) and the TyG index (*r* = 0.278, *P* < 0.001). We also found that the TyG index was positively correlated with HOMA-IR (*r* = 0.328, *P* < 0.001). The 10-year CVD risk score was positively correlated with the TyG index (*r* = 0.348, *P* < 0.001) and HOMA-IR (*r* = 0.142, *P* < 0.001). Logistic regression analysis showed that the TyG index was positively correlated with high baPWV (*OR* = 1.513, 95%*CI* 1.029–2.224, *P* = 0.035) and 10-year CVD risk (*OR* = 2.345, 95%*CI* 1.179–4.667, *P* = 0.015), even after adjusting for confounding factors. Interestingly, after adjusting for HOMA-IR, the association of the TyG index with high baPWV and 10-year CVD risk still existed. However, the association of HOMA-IR with high baPWV (*OR* = 1.022, 95%*CI* 0.994–1.050, *P* = 0.119) and 10-year CVD risk (*OR* = 0.994, 95%*CI* 0.945–1.045, *P* = 0.804) was absent after adjusting for the effects of multiple risk factors.

## Discussion

In this study, for the first time, we demonstrate the association of the TyG index with arterial stiffness and 10-year CVD risk among a larger Chinese population. A major finding of our study was that the TyG index was positively and significantly associated with baPWV as a marker of arterial stiffness and 10-year CVD risk evaluated using Framingham risk score, independent of classical cardiovascular risk factors. Furthermore, our results showed that the association of HOMA-IR with high baPWV and 10-year CVD risk was absent after adjusting for multiple risk factors.

IR is a major characteristic of the pathophysiology of T2DM and plays a crucial role in the pathophysiology of MetS and NAFLD. Previous studies have discovered that a high IR level is not only correlated with an increased risk of developing CVD but is also significantly associated with high risk of cardiovascular outcomes (15, 16). It is well-known that hyperinsulinemic-euglycemic glucose clamp is the gold standard technique for measuring IR, but its clinical application is limited owing to its extended time consumption and high cost. HOMA-IR, which can be calculated from fasting blood glucose and insulin levels, is the most frequently used validated marker of IR in clinical practice. However, plasma insulin levels are usually measured for diabetes mellitus, which are unsuitable for the general population. Hence, multiple surrogate markers of IR have recently emerged, including the TyG index. Moreover, some studies have shown that the predicted TyG index value for IR is better than that of HOMA-IR (17, 18). Several other studies have shown that the TyG index is potentially useful for predicting T2DM and glycemic control in overweight and obese patients with T2DM (8, 19). In addition, the TyG index is superior to HOMA-IR in predicting NAFLD (20). Recently, accumulated epidemiological data indicate that the TyG index is significantly associated with vascular disease. Kim et al. showed that the TyG index is more independently associated with the presence of coronary artery atherosclerosis than HOMA-IR in healthy Korean adults (21). Moreover, subjects with a higher TyG index have a higher risk of developing cardiovascular events and adverse cardiovascular outcomes, independent of confounding factors (22, 23). The TyG index is also a useful marker for predicting the occurrence of subclinical coronary artery disease in the absence of traditional risk factors (24). BaPWV is a simple, non-invasive method, which correlates well with arterial stiffness, and it is also a useful tool for identifying a subgroup in a population that is at increased risk of cardiovascular events (25). However, there are limited studies on the association between the TyG index and baPWV, particularly in a large Chinese population. To the best of our knowledge, there is only one study on the association of the TyG index with baPWV in Shanghai (26), but it was conducted with only older adults (more than 65 years) and did not include the calculation of the cut-off value of the TyG index for high baPWV. In the present study, which was conducted among a relatively high number of apparently healthy adults, we found that the TyG index was significantly correlated with baPWV, even after adjustment for traditional CVD risk factors. Meanwhile, we also found that there was a significant sex-interaction between the TyG index and baPWV. ROC analysis showed that the TyG index had a higher predictive value for high baPWV and in women than men. Therefore, the TyG index was an acceptable predictor of high baPWV only in women, indicating that gender also had a significant effect on the associations between the TyG index and baPWV. Gender differences in the relationship between other risk factors and CVD are now widely accepted. For example, a systematic review and meta-analysis showed that hyperuricemia appears to increase the risk of coronary heart disease incidence and mortality in women, but not in men (27). The mechanisms that cause the TyG index to be less related to high baPWV in men than women remain uncertain and need to be investigated further.

The Framingham risk score is a simplified technique and has been validated across different populations for the assessment of risk level of CVD over 10 years (28, 29). There are limited data on the association between the TyG index and Framingham risk score. The participants in our study were classified as low (< 10%), intermediate (10–20%), and high risk (>20%) of CVD, depending on the Framingham risk score categorization. We found that the percentage of participants in the 10-year CVD risk categories significantly increased with increasing quartiles of the TyG index. Moreover, the TyG index was significantly correlated with intermediate- or high-risk categories of Framingham risk score after adjusting for confounding factors. ROC analysis showed that the AUROC of the TyG index for predicting the 10-year CVD risk was 0.778, with a sensitivity of 71.2% and a specificity of 74.3% in women. However, the AUC value of the TyG index for predicting 10-year CVD risk in men was relatively smaller, which indicated that the TyG index was an acceptable predictor of 10-year CVD risk only in women. In addition, the AUROC of TyG-baPWV for predicting the 10-year CVD risk was larger than those of the TyG index and baPWV, respectively, in women, indicating that combination the TyG index and baPWV could better predict cardiovascular risk.

HOMA-IR is another surrogate marker of IR. The association of HOMA-IR with CVD has been studied widely. https://www.ncbi.nlm.nih.gov/pubmed/?term=Kim%20MK%5BAuthor%5D&cauthor=true&cauthor_uid=28830471 et al. reported that the TyG index was more independently associated with the presence of coronary artery atherosclerosis than HOMA-IR (21). Irace et al. found that the TyG index was better associated with carotid atherosclerosis than HOMA-IR (30). Similar results were also observed in our study. In this study, we found that after adjusted for traditional CVD risk factors, the TyG index was still correlated with baPWV and 10-year CVD risk, while HOMA-IR was not, which may be explained by the fact that the two indices reflect different aspects of IR. The TyG index reflects IR mainly in the muscles, whereas HOMA-IR reflects IR mainly in the liver (31, 32). Hence, we speculated that peripheral IR might have contributed to the pathophysiology of CVD.

This study has several limitations. First, it is difficult to determine whether the TyG index has a causative effect on arterial stiffness because of the cross-sectional design of this study. Second, we could not collect data on alcohol consumption, nutritional diet, and exercise habits of the participants, which could affect the circulating TG levels. Third, we did not determine serum insulin levels in all participants and the lack of the association of HOMA-IR with baPWV and 10-year CVD risk may be attributed to the small number of patients with HOMA-IR data. Fourth, the participants in this study were enrolled at one health promotion center, and therefore, the generalizability of the results may be limited.

## Conclusion

Our study demonstrates that the TyG index is independently associated with arterial stiffness measured through baPWV and 10-year CVD risk evaluated using Framingham risk score among a sample of apparently healthy Chinese population. This study shows the promising value of the TyG index as an accessible biomarker for baPWV and 10-year CVD risk in women only. It is important to closely examine people whose TyG indexes are elevated, as lifestyle modification and appropriate treatment, are essential strategies in preventing future CVD among them.

## Data Availability Statement

The original contributions presented in the study are included in the article/Supplementary Material, further inquiries can be directed to the corresponding author/s.

## Ethics Statement

The studies involving human participants were reviewed and approved by the Ethical Committee of the First Affiliated Hospital of Nanjing Medical University. The patients/participants provided their written informed consent to participate in this study.

## Author Contributions

WG and QZ participated in the study design, wrote and modified the manuscript, and prepared tables and figures. WG, WZ, JW, XL, JL, PQ, CZ, and NX were involved in the conduct of the study and data collection. WG and WZ made contributions to data analysis and results interpretation. All authors contributed to the article and approved the submitted version.

## Conflict of Interest

The authors declare that the research was conducted in the absence of any commercial or financial relationships that could be construed as a potential conflict of interest.
