# Supplementary material for: Triglyceride Glucose Index Is Associated With Arterial Stiffness and 10-Year Cardiovascular Disease Risk in a Chinese Population
Source: Front Cardiovasc Med. 2021 Mar 19;8:585776. doi: 10.3389/fcvm.2021.585776 (PMC8017152; doi:10.3389/fcvm.2021.585776)
Supplement: Supplementary file 1 [file Data_Sheet_1.PDF]

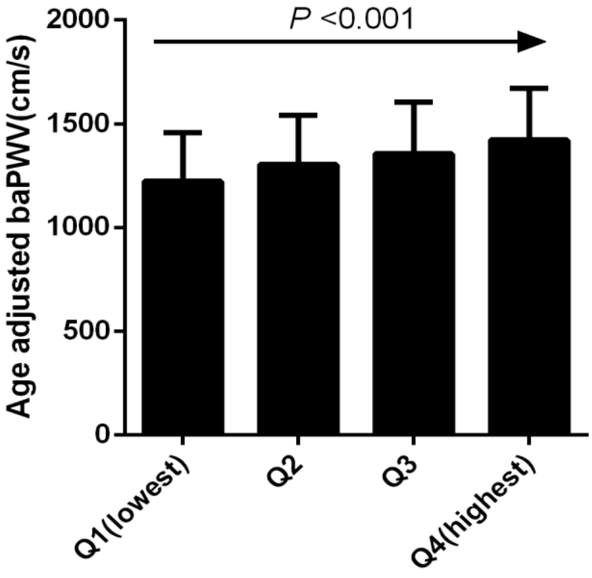

Supplementary Figure1. Age adjusted BaPWV compared across the TyG index quartiles for non-obese participants

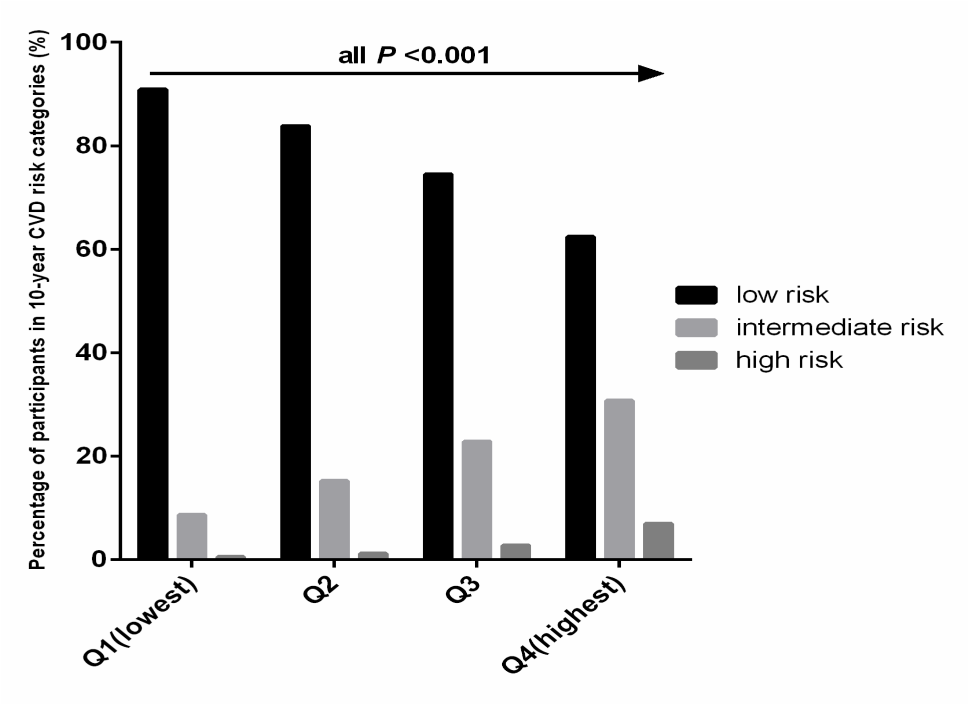

Supplementary Figure2. Percentages of 10-CVD risk categorise compared across the quartiles of the TyG index in non-obese participants

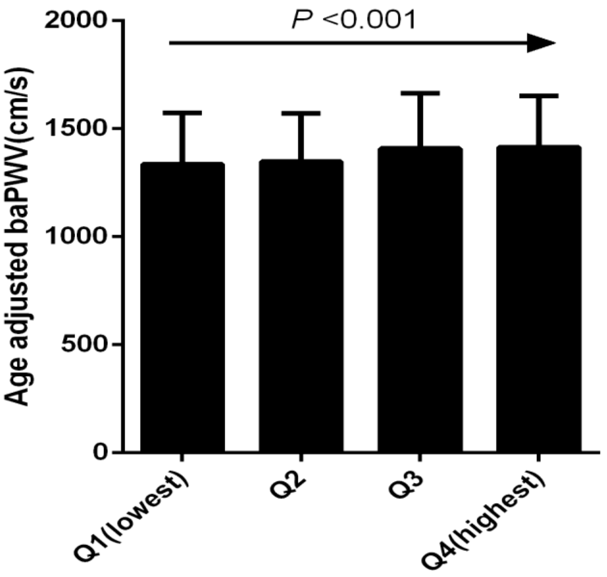

Supplementary Figure3. Age adjusted BaPWV compared across the TyG index quartiles for obese participants

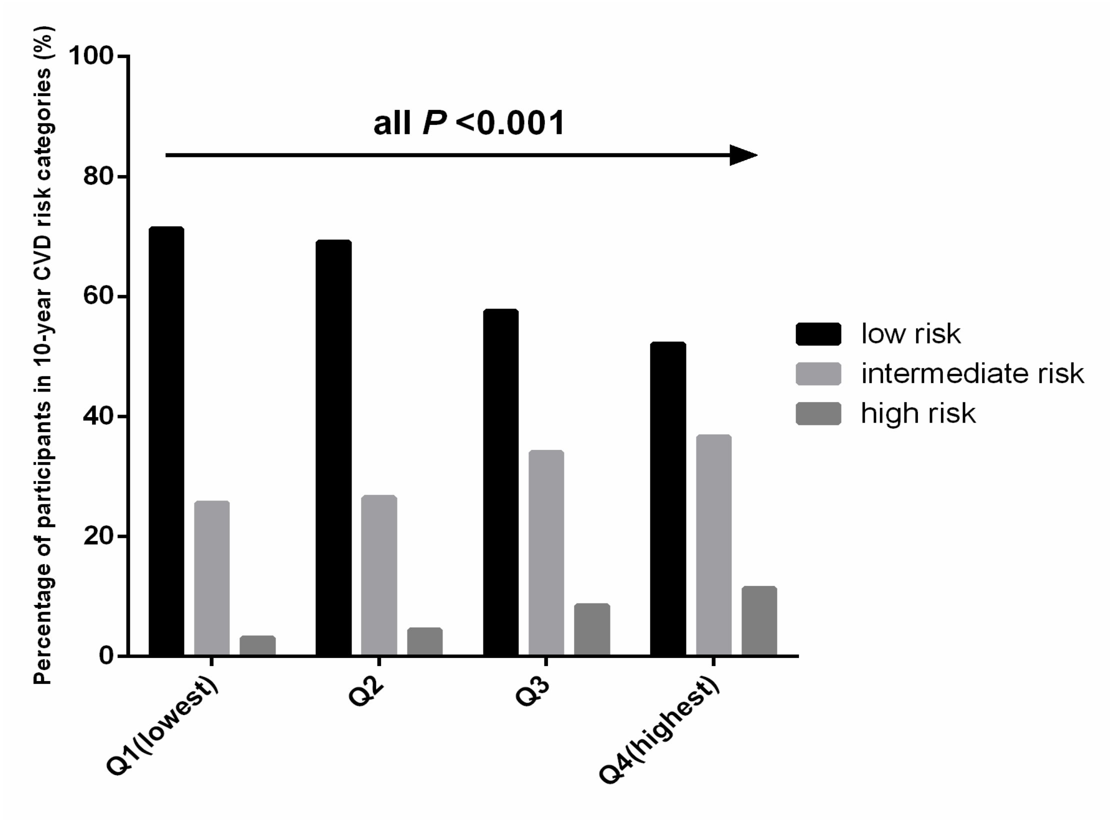

Supplementary Figure2. Percentages of 10-CVD risk categorise compared across the quartiles of the TyG index in obese participants

Supplementary Table1 Impact of TyG index on high baPWV according to obesity

	Non obesity		Obesity	
Model	OR (95%*CI*)	*P*	OR (95%*CI*)	*P*
1	2.315(2.157-2.486)	<0.001	1.710(1.413-2.069)	<0.001
2	1.788(1.620-1.973)	<0.001	1.420(1.207-1.672)	<0.001

Model1: unadjusted

Model2: adjustment for age, smoking, BMI, Pulse pressure, HbA1c, TC, LDL-C, HDL-C and uric acid

Supplementary Table2 Impact of TyG index on the 10-year CVD risk according to obesity

	Non obesity		Obesity	
Model	OR (95%*CI*)	*P*	OR (95%*CI*)	*P*
1	2.315(2.157-2.486)	<0.001	1.762(1.152-2.053)	<0.001
2	1.609(1.462-1.769)	<0.001	1.388(1.160-1.661)	<0.001

Model1: unadjusted

Model2: adjustment for BMI, Pulse pressure, HbA1c, LDL-C, and uric acid
